# Culture of human mesenchymal stem cells using a candidate pharmaceutical grade xeno-free cell culture supplement derived from industrial human plasma pools

**DOI:** 10.1186/s13287-015-0016-2

**Published:** 2015-03-13

**Authors:** José M Díez, Ewa Bauman, Rodrigo Gajardo, Juan I Jorquera

**Affiliations:** Cell Culture and Virology Laboratory, Research & Development Biologics Industrial Group. Grifols, Carrer Llevant, 11, 08150 Parets del Vallès, Barcelona, Spain

## Abstract

**Introduction:**

Fetal bovine serum (FBS) is an animal product used as a medium supplement. The animal origin of FBS is a concern if cultured stem cells are to be utilized for human cell therapy. Therefore, a substitute for FBS is desirable. In this study, an industrial, xeno-free, pharmaceutical-grade supplement for cell culture (SCC) under development at Grifols was tested for growth of human mesenchymal stem cells (hMSCs), cell characterization, and differentiation capacity.

**Methods:**

SCC is a freeze-dried product obtained through cold-ethanol fractionation of industrial human plasma pools from healthy donors. Bone marrow-derived hMSC cell lines were obtained from two commercial suppliers. Cell growth was evaluated by culturing hMSCs with commercial media or media supplemented with SCC or FBS. Cell viability and cell yield were assessed with an automated cell counter. Cell surface markers were studied by indirect immunofluorescence assay. Cells were cultured then differentiated into adipocytes, chondrocytes, osteoblasts, and neurons, as assessed by specific staining and microscopy observation.

**Results:**

SCC supported the growth of commercial hMSCs. Starting from the same number of seeded cells in two consecutive passages of culture with medium supplemented with SCC, hMSC yield and cell population doubling time were equivalent to the values obtained with the commercial medium and was consistent among lots. The viability of hMSCs was higher than 90%, while maintaining the characteristic phenotype of undifferentiated hMSCs (positive for CD29, CD44, CD90, CD105, CD146, CD166 and Stro-1; negative for CD14 and CD19). Cultured hMSCs maintained the potential for differentiation into adipocytes, chondrocytes, osteoblasts, and neurons.

**Conclusions:**

The tested human plasma-derived SCC sustains the adequate growth of hMSCs, while preserving their differentiation capacity. SCC can be a potential candidate for cell culture supplement in advanced cell therapies.

## Introduction

Human mesenchymal stem cells (hMSCs) are multipotent cells with the capacity to differentiate into multiple types of functionally mature lineage-specific cells [1,2]. In addition, hMSCs have a low immunogenicity – which can help to improve allogenic transplantation and avoid immune rejection, one of the main complications of stem cell use in cell therapy [3-8]. These characteristics make hMSCs of great interest for use in regenerative medicine therapies, as well as a treatment for many diseases [9]. The infusion of hMSCs has been positively tested in preclinic acute lung injuries, myocardium stroke, diabetes, and multiple sclerosis, as well as hepatic and renal failure, among other areas [10,11].

Representing the majority of the adherent fraction of the bone marrow, hMSCs are 0.001 to 0.01% of the whole marrow and can be isolated easily from different tissues [12]. Although *in vivo* sources provide very small quantities of hMSCs, these cells can be expanded by culture *in vitro*. However, the need for a stable, standard, and safe *in vitro* culture medium is one of the major shortcomings of hMSC use in advanced therapies.

hMSCs are usually cultured in media supplemented with fetal bovine serum (FBS), which provides growth factors, adhesion factors, and vital nutrients essential for the culture of this type of cell [13,14]. The use of FBS (or nonxeno-free supplements) is appropriate for *in vitro* research but, due to its animal origin, could be a concern if the cultured cells are used for human cell therapy [15-17]. Furthermore, the use of xenogeneic sera has high lot-to-lot variability and is associated with potential patient immune reactions [18]. Therefore, for the translation of stem cells to clinical uses, it is ideal to perform the culture under xeno-free conditions [16].

A number of studies have tested alternative cell culture serum supplements from various human origins to determine a better substitute for FBS [15,16,19-21]. There is consensus that the use of human-derived serum supplements is the preferred alternative in cell cultures intended for cell-based therapy. However, blood bank products have small pool sizes that could provide less consistency than products from a larger, industrial plasma pool.

In an effort to overcome these shortcomings, a new industrial, good manufacturing practice -produced, xeno-free, pharmaceutical-grade, human plasma-derived supplement for cell culture (SCC) is under development at Grifols (Parets del Vallès, Barcelona, Spain). SCC has been successfully used for the culture of human embryonic stem cells, for the culture of induced pluripotent stem cells, for the reprogramming of human fibroblasts to induced pluripotent stem cells [22], and for the culture of other mammalian cell lines [23]. In the present study, SCC was tested to determine its ability to support cell growth of hMSCs *in vitro*. In addition, characterization and viability of hMSCs cultured in SCC-containing media as well as hMSC capacity to differentiate into neural precursors were assessed.

## Methods

### Production of the human cell culture supplement

SCC is a freeze-dried preparation manufactured under good manufacturing practice rules and is obtained from human plasma through cold-ethanol industrial fractionation [24]. Plasma used for SCC production is collected from healthy donors after signing the consent agreement for automatic plasmapheresis at US Food and Drug Administration-licensed plasmapheresis centers of the Grifols network (Biomat, USA), where plasma is specifically collected for use as the starting material in the industrial production of plasma-derived therapeutic products. Fractionation plasma pools contain donations from over 1,000 different donors. Each individual donation is serologically tested for transfusion-transmissible viral markers, and all plasma is tested using nucleic acid techniques for the presence of DNA or RNA from human immunodeficiency virus, hepatitis A virus, hepatitis B virus, hepatitis C virus, and human parvovirus B19. Moreover, SCC has a specific viral inactivation step (gamma irradiation) incorporated into the production process in addition to purification steps with pathogen removal capacity. Owing to manufacturing conditions, SCC biological properties are consistent from lot to lot.

For the experiments described in this study, if not stated otherwise, SCC was reconstituted with Basal Medium 2 (BM2), a culture medium developed at Grifols. The BM2 formulation is based on Dulbecco’s modification of Eagle’s medium and Ham’s F12 (Gibco-Life Technologies, Carlsbad, CA, USA) with commonly employed growth factors (such as basic fibroblast growth factor plus the growth factors from platelet lysates) and other elements added (such as insulin, sodium selenite, and ethanolamine). This medium can be used in combination with SCC and FBS.

### Human mesenchymal stem cell culture

Two hMSC cell lines obtained from the bone marrow of healthy donors, provided by two different commercial suppliers – PT-2501 from a 21-year-old female and a 22-year-old female (Lonza Group Ltd, Basel, Switzerland), and C-12974 from a 64-year-old male (PromoCell GmbH, Heidelberg, Germany) – were used in this study. Because these cells are commercially available, no patient consent or approval from Ethics Committee was needed.

Cells were cultured at 37°C and 8% carbon dioxide with the media recommended by the manufacturer. The media were changed every other day and the cells were split enzymatically with Trypsin: 1× phosphate-buffered saline (Ref. 14190–185; Gibco Life Technologies) and Tryple Express (Ref. 12604–013; Gibco-Life Technologies) for PT-2501 cells, and the Trypsin kit (PromoCell GmbH) for C-12974 cells. Cultures were trypsinized when the cells were approximately 90% confluent.

### Human mesenchymal stem cell growth evaluation

Cell growth of hMSCs was qualitatively evaluated by microscopy and classified as positive (+) for positive growth to reach 90% confluence and negative (−) for no growth (<400 cells/cm^2^) in five different media formulations: BM2 without supplementation, BM2 with FBS 10% (mesenchymal stem cell qualified; Gibco-Life Technologies), BM2 containing SCC up to 40% v/v, and media from each of the two hMSC suppliers (Lonza Group and PromoCell). The same commercial cells were also grown in a classical medium (Dulbecco’s Modification of Eagle’s Medium and Ham’s F12) supplemented with FBS.

For the determination of the working SCC concentration required for the optimal hMSC growth in BM2, SCC concentrations from 0 to 40% v/v were tested. The extent of cell proliferation was determined by a tetrazolium-based colorimetric assay for cell chemosensitivity that detects metabolically active cells [25]. The Cell Proliferation Assay kit (Merck Millipore, Billerica, MA, USA) was used, following the instructions of the manufacturer. Briefly, prepared media were filtered through 0.45 μm and 0.22 μm pore filters (Merck Millipore); cells were trypsinized, resuspended in their medium, and seeded at a concentration of 5,000 cells/cm^2^ in plates and flasks (Corning Inc. Life Sciences, Tewksbury, MA, USA). Cells were incubated at 37°C and 5% carbon dioxide without changing the media. After 5 days incubation, 20 μl WST-1/ECS reagent (Ref. 2210; Merck Millipore) was added. After a subsequent 4 hours of incubation, absorbance at 450 nm was read.

### Human mesenchymal stem cell culturing assessments

Studies on hMSC yield, replicative capacity, and cell viability were performed with the commercial hMSC lines in two consecutive culture passages from seeding (passages 1 and 2; inoculum with a fixed cell count at day 0) after 7 to 10 days in culture. Cultured cells in commercial medium and medium containing SCC at the determined working concentration for optimal hMSC growth were compared (Student’s *t* test in three experiments each; mean ± standard deviation).

Cell yield was assessed by microscopy and cell count. When hMSCs reached 90% confluence, cells were trypsinized. A cell count was then performed by an automated cell counter (Countess™; Invitrogen-Life Technologies, Carlsbad, CA, USA). The result was expressed as the number of cells per square centimeter of cell culture surface (cells/cm^2^). Cell viability was assessed after trypsinization of the culture flask using trypan blue staining (Life Technologies, Carlsbad, CA, USA). The number of viable cells in relation to the total cell count was calculated [25].

Cell replicative capacity was evaluated by the population doubling time (PDT) [26]. The hMSCs at a density of 5,000 cells/cm^2^ were seeded and cultured as described. Cell numbers were calculated with the following formula [27]:

$$ \mathrm{P}\mathrm{D}\mathrm{T} = 1\ /\ \left[3.32\ \left( \log\ {\mathrm{N}}_{\mathrm{H}}\hbox{--}\ \log\ {\mathrm{N}}_1\right)\ /\ \left({\mathrm{t}}_2\hbox{--}\ {\mathrm{t}}_1\right)\right] $$

where N_H_ is the number of harvested cells at the end of the growth period, N_1_ is the number of seeded cells, t_1_ is the time at seeding, and t_2_ is the time elapsed between t_1_ and cell harvesting.

### Characterization of hMSC cell surface markers

Immunofluorescence staining was used to qualitatively determine cell surface markers in the hMSC lines cultured in medium containing SCC (at the determined working concentration for optimal hMSC growth). Seven positive markers – CD29 (Ref. 303002; Biolegend, San Diego, CA, USA), CD44 (Ref. CBL154; Merck Millipore ), CD90 (Ref. CBL1500F; Merck Millipore), CD105 (Ref. MABT117; Merck Millipore), CD146 (Ref. EPR3208; Merck Millipore), CD166 (Ref. 343902; Biolegend), and Stro-1 (Ref. MAB4315; Merck Millipore) – and two negative markers – CD14 (Ref. MAB1794; Merck Millipore) and CD19 (Ref. MAB1794; Merck Millipore) – for hMSCs were studied [12,28-33]. These markers were also studied for cells cultured with commercial media (data not shown).

When the hMSCs were at day 6 and at day 7 of culture, a plate was seeded with 1.9 × 10^4^ cell/ml and incubated for 3 days at 37°C and 5% carbon dioxide.

The cells were fixed with 4% paraformaldehyde (Ref. FB001; Invitrogen-Life Technologies) at room temperature for 40 minutes, followed by overnight incubation with a blocking solution. Fixed cells were then incubated overnight at 4°C with the primary antibodies (diluted 1:100 in blocking buffer) against the markers. Afterwards, the sample was washed with phosphate-buffered saline containing blocking buffer and incubated with secondary antibodies (diluted 1:100 in blocking buffer), donkey anti-mouse IgG conjugated with fluorescein isothiocyanate (Ref. AP192F;Merck Millipore), and goat anti-mouse IgM conjugated with Cy3 (Ref. AP128C; Merck Millipore). Finally, the sample was counterstained with 4',6-diamidino-2-phenylindole (Ref. D3571; Invitrogen-Life Technologies) and visualized under the fluorescence microscope (Axiobserver LD Plan-Neofluar objective; Carl Zeiss, Göttingen, Germany).

### Differentiation assays

#### Adipogenic differentiation

The hMSCs were cultured in commercial medium or one of two laboratory-made media formulations supplemented with SCC – each containing additional platelet lysate. After reaching 90% confluence, the cells were harvested and reseeded into a 24-well plate (6 × 10^4^ cells/well) for adipogenic differentiation. Commercial differentiation medium (Ref. C-28011; PromoCell) was used to induce adipogenesis. After 14 days, differentiation was assessed by observation of cell morphology and the specific staining of lipid droplets with Oil Red O (Ref. O0625-25G; Sigma-Aldrich, St. Louis, MO, USA).

#### Osteogenic differentiation

Similarly, the hMSCs were cultured in commercial medium or one of two laboratory-made media formulations supplemented with SCC. After reaching 90% confluence, the cells were harvested and reseeded into a 24-well plate (6 × 10^4^ cells/well) for osteogenic differentiation. The hMSCs were differentiated into osteoblasts using a commercial differentiation medium (Ref. C-28013; PromoCell). After 21 days of incubation, differentiation was assessed by observation of cell morphology changes and by the specific staining of extracellular calcium deposits with Alizarin Red S (Ref. A5533-25G; Sigma-Aldrich). Moreover, alkaline phosphatase activity was measured with the use of the combination of nitro-blue tetrazolium chloride and 5-bromo-4-chloro-3’-indolyl phosphate p-toluidine salt and nitro-blue tetrazolium chloride (BCIP/NBT tablet, Ref. B5655-5TAB; Sigma-Aldrich).

#### Chondrogenic differentiation

The hMSCs were cultured in commercial medium or one of two laboratory-made media formulations supplemented with SCC. After reaching 90% confluence, the cells were harvested and reseeded in 15 ml conical tubes at 2.5 × 10^5^ cells/tube. Mesenchymal stem cell (MSC) spheroids formed in the tubes and were incubated with commercial MSC chondrogenic differentiation medium (Ref. C-28012; PromoCell). After 21 days of culture, the cells were stained with Alcian Blue (Ref. A3157-10G; Sigma-Aldrich) to determine differentiation.

#### Neurogenic differentiation

Neurogenic cell differentiation studies were also performed with the commercial hMSC lines. Cultured cells in commercial medium and in BM2 + SCC medium (at the determined optimal concentration for hMSC growth) were morphologically compared. Plates were covered with 10 μg/ml fibronectin (Ref. C-43050; PromoCell) in 1× phosphate-buffered saline and left to dry. The hMSCs were seeded at a concentration of 4,000 cells/cm^2^ and cultured until cells were approximately 50 to 90% confluent. Then, hMSC neurogenesis was induced by switching the medium to a commercial differentiation medium (Ref. C-28015; PromoCell). Differentiation and cell morphology (acquisition of mature axonal/dendritic polarity) was assessed by the specific staining of Nissl bodies [34] and microscopy [35].

## Results

### Cell growth

The hMSCs grew to confluence with both the commercial media and the BM2 medium supplemented with SCC or FBS (Table 1). The BM2 medium without supplementation did not promote cell growth. The cells also did not grow when Dulbecco’s Modification of Eagle’s Medium and Ham’s F12 supplemented with FBS only was used.

**Table 1:** **Cell growth of the human mesenchymal stem cells cultured in different media formulations and supplements**

**hMSC line**	**Medium**	**Supplement**	**Growth**
PT-2501	Commercial	Commercial	(+)
	BM2	FBS	(+)
		SCC	(+)
		None	(−)
	DMEM/F12	FBS	(−)
		SCC	(−)
C-12974	Commercial	Commercial	(+)
	BM2	FBS	(+)
		SCC	(+)
		None	(−)
	DMEM/F12	FBS	(−)
		SCC	(−)

BM2, Basal Medium 2; DMEM/F12, Dulbecco’s Modification of Eagle’s Medium and Ham’s F12; FBS, fetal bovine serum; hMSC, human mesenchymal stem cell; SCC, supplement for cell culture.

**Figure 1:**
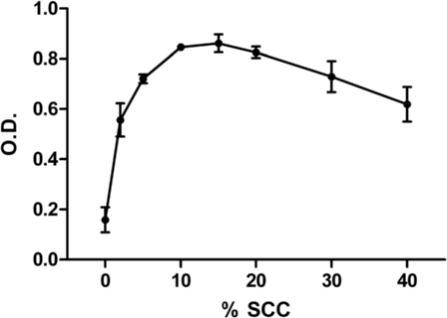
**Human mesenchymal stem cell proliferation in Basal Medium 2 culture medium containing supplement for cell culture.** Proliferation was determined by colorimetric assay (absorbance at 450 nm) for cell chemosensitivity to detect metabolically active cells. Concentrations of supplement for cell culture (SCC) ranged from 0 to 40% v/v. O.D., optical density.

Results of the studies to determine the SCC concentration for optimal hMSC growth are shown in Figure 1. Maximal cell growth was observed between 10 and 20% v/v. Therefore, 15% v/v was used as the working SCC concentration for subsequent experiments.

### Cell yield

**Figure 2:**
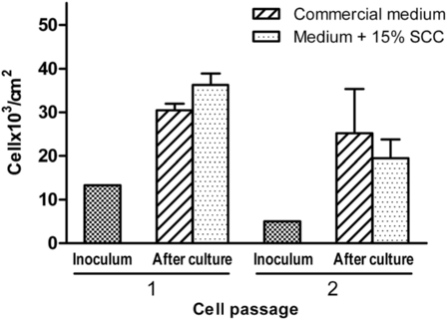
**Human mesenchymal stem cell yield in two consecutive culture passages.** Cells were maintained for 7 to 10 days in culture with commercial medium and with medium containing 15% supplement for cell culture (SCC) from seeding with a fixed cell count at day 0 (inoculum) (*n* = 3).

The hMSC yield in the two passages of culture with medium containing 15% SCC was similar to the cell yield obtained with the commercial medium (Figure 2). Starting from the same cell seeding load (inoculum), the average yield was 2.3-fold to 2.7-fold for passage 1 and fourfold to fivefold for passage 2.

### Cell replicative capacity

The PDT was similar for both the commercial medium and the medium containing 15% SCC. At passage 1, the average PDT was 4.5 ± 0.7 days for the commercial medium and 4.0 ± 1.1 days for the medium containing 15% SCC. At passage 2, the PDT was 4.2 ± 2.2 days and 5.8 ± 1.6 days for the commercial medium and the medium containing SCC, respectively.

### Cell viability

Cell viability was high for both media, with averages of 95.5 ± 1.8% and 92.2 ± 6.3% (passages 1 and 2, respectively) for cells cultured with the commercial medium and 96.5 ± 3.1% and 92.2 ± 4.3% (passages 1 and 2, respectively) for cells cultured with medium containing 15% SCC.

### Cell surface markers

**Figure 3:**
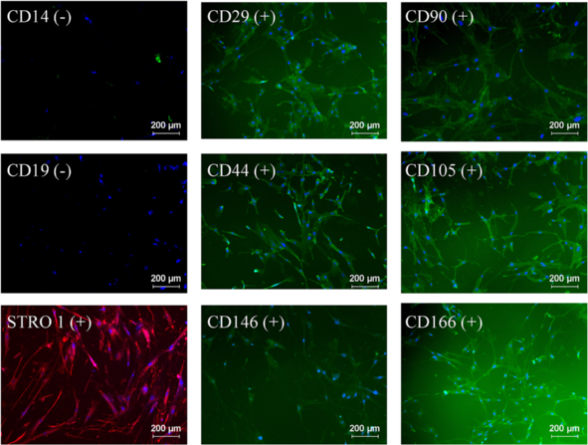
**Surface markers.** Illustrative micrographs of human mesenchymal stem cell surface markers as determined by immunofluorescence staining in cells cultured in medium containing 15% supplement for cell culture.

The hMSCs cultured in medium containing 15% SCC were positive for CD29, CD44, CD90, CD105, CD146, CD166, and Stro-1, whereas they were negative for CD14 and CD19 (Figure 3).

### Adipogenic differentiation

**Figure 4:**
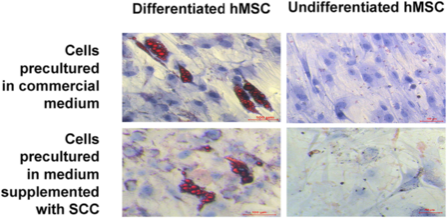
**Adipogenic differentiation.** Illustrative micrographs of human mesenchymal stem cells (hMSCs) cultured with commercial medium and with Basal Medium 2 with 15% supplement for cell culture (SCC), showing adipogenic differentiation. Lipid droplets (oil Red O staining) characteristic for adipocytes can be observed.

The hMSCs cultured with SCC preserved the potential for differentiation into adipocytes. After adipogenic differentiation, hMSCs showed a morphology characteristic for adipocytes (Figure 4). Oil Red O staining revealed lipid droplets. In all of the cell groups, incubated in either commercial basal medium or BM2 formulations supplemented with SCC and platelet lysate, differentiation was observed.

### Osteogenic differentiation

**Figure 5:**
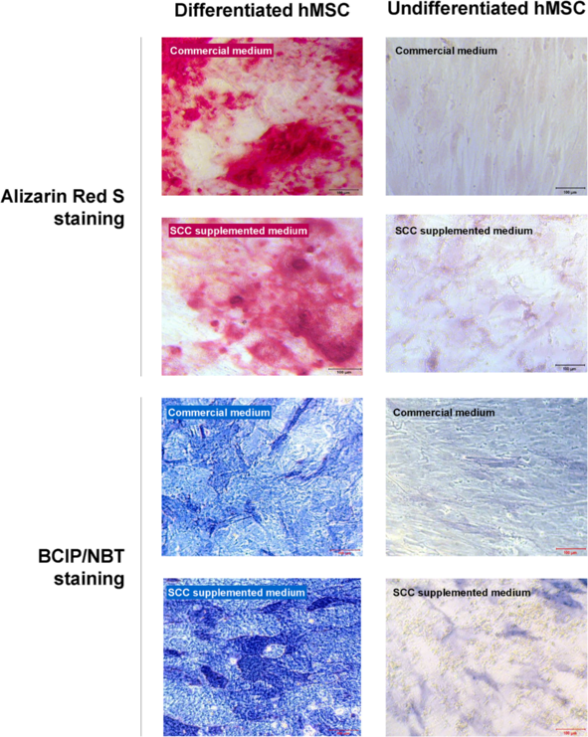
**Osteogenic differentiation.** Illustrative micrographs of human mesenchymal stem cells (hMSCs) cultured with commercial medium and with Basal Medium 2 with 15% supplement for cell culture (SCC), showing osteogenic differentiation. Alizarin Red S staining revealed extracellular calcium deposits. Alkaline phosphatase activity was present in the differentiated cells, which was shown by 5-bromo-4-chloro-3'-indolyl phosphate p-toluidine salt and nitro-blue tetrazolium chloride (BCIP/NBT tablet, Ref. B5655-5TAB; Sigma-Aldrich, St. Lous, MO, USA) staining.

The hMSCs cultured with SCC preserved the potential for differentiation into osteogenic cells (Figure 5). After inducing differentiation, the cells showed traits characteristic for osteoblasts – cuboidal shape with flattened morphology. Alizarin Red S staining revealed extracellular calcium deposits. Elevated alkaline phosphatase activity was present in the differentiated cells, which was shown by the BCIP/NBT tablet staining. In all of the cell groups, incubated in either commercial MSC basal medium or BM2 formulations supplemented with SCC, differentiation was observed at a similar level.

### Chondrogenic differentiation

**Figure 6:**
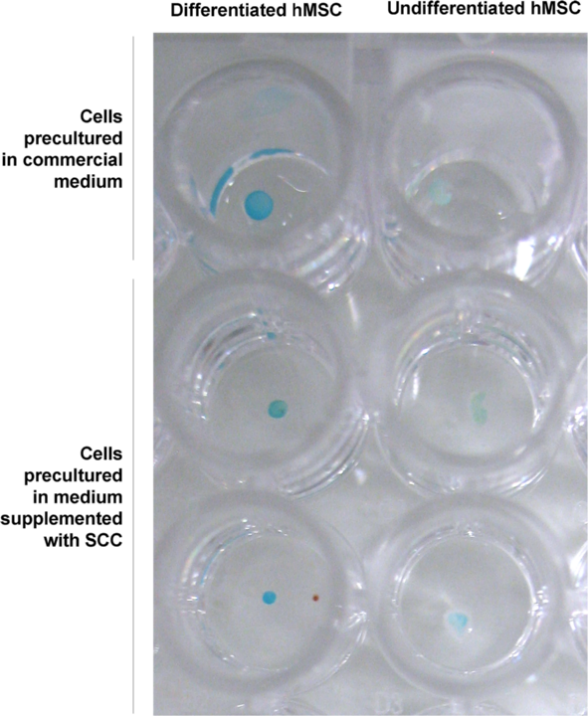
**Chondrogenic differentiation.** Macroscopic photograph showing human mesenchymal stem cells (hMSCs) cultured with commercial medium and with Basal Medium 2 with 15% supplement for cell culture (SCC), brought under chondrogenic differentiation. Pellets were stained with Alcian Blue for cartilage. Differentiated spheroids show intense blue color, while undifferentiated mesenchymal stem cells are faintly bluish or almost colorless.

The hMSCs cultured with SCC preserved the potential for differentiation into cartilage (Figure 6). After inducing differentiation, Alcian Blue staining revealed the presence of glycosaminoglycans characteristic for the extracellular matrix of cartilage. In all of the cell groups, incubated in either commercial MSC basal medium or BM2 formulations supplemented with SCC, differentiation was observed at a similar level.

### Neurogenic differentiation

**Figure 7:**
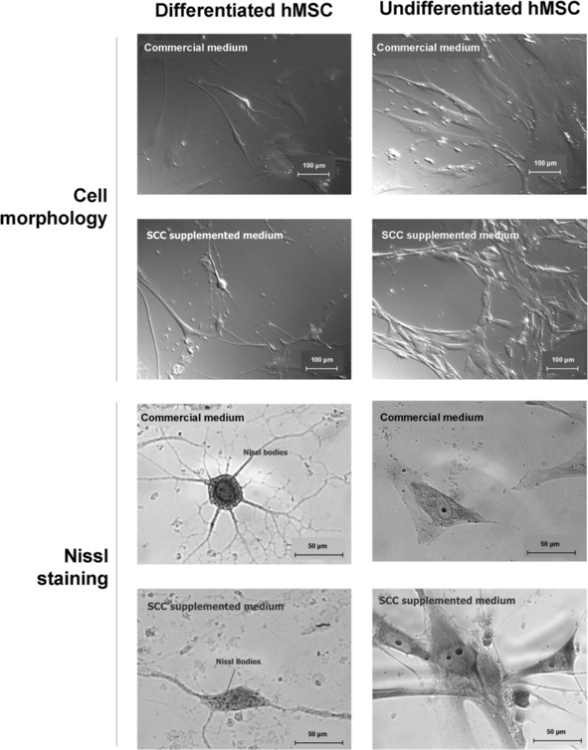
**Neurogenic differentiation.** Illustrative micrographs of human mesenchymal stem cells (hMSCs) cultured with commercial medium and with Basal Medium 2 with 15% supplement for cell culture (SCC), before and after induction of neurogenic differentiation. Elongated axons and dendrites as well as stained Nissl bodies can be observed in the differentiated cells.

After neurogenic differentiation induction, microscopy examination of hMSCs cultured with both the commercial medium and with medium containing 15% SCC showed neuronal morphology with elongated axons and dendrites (Figure 7). These features were not observed in the undifferentiated cells. Nissl staining showed Nissl bodies around the nucleus in differentiated cells but not in undifferentiated cells.

## Discussion

The interest in hMSCs for use in cell therapy and tissue engineering has been growing in the last two decades. Because the use of animal sera to supplement hMSC culture media may be problematic, several studies have recently addressed the use of xeno-free [36-40] and serum-free [38,40-42] culture conditions for isolation/expansion of hMSCs from several sources such as the bone marrow [36,39,41,42], adipose tissue [36,38,39], human Warton’s jelly [37], and umbilical cord matrix [40]. In this study, we have evaluated the first xeno-free, pharmaceutical-grade SCC derived from human plasma, currently under development. Our study has shown that SCC can be a viable substitute for FBS in clinical-grade, human stem cell culture.

The tested SCC is a freeze-dried product manufactured under good manufacturing practice, ensuring consistency and compliance because all critical processes are clearly defined, controlled, and validated. In addition, lyophilization is convenient for transport and storage. SCC derived from an industrial plasma pool constituted from over 1,000 human donors results in a consistent final product with little variability between batches [43]. High lot-to-lot stability is evidenced by the low standard deviation (below one-third of the mean in average) observed in our studies on hMSC yield, replicative capacity, and cell viability. SCC also provides factors, which could facilitate the improved growth of cells under conditions more similar to those that occur within the human body. SCC can therefore help to overcome the problem of extensive lot-to-lot variability associated with FBS due to its complex mixture of defined and unknown biological products [44].

Cell growth tests from this study highlight the potential of SCC as media supplementation for not only hMSC culture. As reported previously, SCC as a media supplement can also be used for other cell types, including human embryonic stem cells, induced pluripotent stem cells, Chinese hamster ovarian cells, Vero cells, and mouse BALB/c myeloma cells, as reported previously [22,23]. With 15% SCC supplementation, the hMSC yield, replicative capacity, and cell viability were comparable with those observed with the commercial medium, indicating that normal use of SCC as a substitute for FBS can be performed reliably. A previous study has also tested cell growth and differentiation capacity with various concentrations of autologous serums versus FBS [45].

Although no definitive characteristic marker has been identified for hMSCs, there are a number of surface receptors associated with their function, which helps to characterize hMSCs. The International Society for Cellular Therapy has proposed that MSCs are positive for antigens such as CD90 (Thy-1) and CD105 (endoglin) [29], which were detected in our studies. Additionally, these cells do not express hematopoietic markers such as CD14 and B-cell markers such as CD19 [29]. Consistent with these studies, our cultured hMSCs did not express such antigens. Other surface receptors detected in our cultured hMSCs included CD44 and CD29 (integrin β1), which are involved in cell–matrix interactions and have been shown to be expressed in MSCs [28,31]. In addition, CD146 (MCAM), CD166 (ALCAM), and Stro-1 were expressed in our cultured hMSCs. These antigens can be employed as phenotypic markers for MSCs, although they can also be found in other cell types [12,32,33]. Ultimately for the cell surface markers studied, hMSCs cultured with SCC-supplemented medium showed the cell phenotype expected for undifferentiated hMSCs, at least for the first two culture passages. Currently, similar results have been obtained in preliminary studies performed in our laboratory in which hMSCs from other origins exhibit similar growth and cell surface marker expression (data not shown).

The capacity of the cultured hMSCs to maintain their multipotent state to further differentiate into other cells types is a key issue for clinical therapies. According to Dominici and colleagues, the minimal criteria for hMSC to be defined as such are that the cells have to be able to differentiate into osteogenic, adipogenic, and chondrogenic cell lines [29]. The hMSCs cultured in the medium supplemented with SCC maintained the capacity to differentiate into adipocytes, osteoblasts, and chondrocytes, indicating that their multipotent potential is preserved by SCC supplementation. This is particularly important, as hMSC differentiation potential is crucial for implementation in any clinical applications, including regenerative medicine therapies.

Since the central nervous system has limited capacity of regenerating lost neurons, transplantation of pluripotent stem cells or stem cell-derived progenitors is seen as a promising therapeutic strategy to repair the damaged tissue in neurological conditions such as Parkinson’s disease, Alzheimer’s disease, Huntington’s disease, stroke, or spinal cord injury [46]. The differentiation of cultured hMSCs into cells of neuronal lineages can be monitored through follow-up of changes in cell morphology such as the formation of dendrites and axons [35]. Alternatively, structures specific to the soma of neurons such as Nissl bodies can be detected immunohistochemically [34]. In our study, these features were observed in hMSCs after neurogenic differentiation induction. Therefore, hMSCs cultured with commercial medium or SCC-supplemented medium preserved the potential for differentiation into neurons.

The use of commercial hMSCs instead of cells isolated directly from donors is a limitation of the study when considering clinical implications. In addition, evaluations were mostly qualitative and were performed in a limited number of culture passages. To confirm SCC clinical applicability, further investigations such as genotyping assessments are advisable.

## Conclusions

Grifols’ human plasma-derived SCC is a safe and stable xeno-free, pharmaceutical-grade supplement that sustains the growth of hMSCs. The hMSCs cultured with SCC are able to later differentiate into a specific cell type. These features make this human plasma-derived SCC a potential candidate for cell culture supplementation in advanced cell therapies designated for regenerative medicine applications.

## Abbreviations

BM2: Basal Medium 2
FBS: fetal bovine serum
hMSC: human mesenchymal stem cell
MSC: mesenchymal stem cell
PDT: population doubling time
SCC: supplement for cell culture
